# The Efficacy of Triamcinolone Acetonide in Keloid Treatment: A Systematic Review and Meta-analysis

**DOI:** 10.3389/fmed.2016.00071

**Published:** 2016-12-27

**Authors:** Thian-Sze Wong, John Zeng-Hong Li, Siqi Chen, Jimmy Yu-Wai Chan, Wei Gao

**Affiliations:** ^1^Department of Surgery, The University of Hong Kong, Pokfulam, Hong Kong; ^2^Department of Otolaryngology, The First People’s Hospital of Foshan, Foshan, Guangdong, China

**Keywords:** controlled trial, keloid, meta-analysis, treatment modalities, triamcinolone acetonide

## Abstract

Keloid is a cutaneous dermal outgrowth resulting from uncontrolled deposition of collagen and glycosaminoglycan around the wound. The uncontrolled and persistent growth of keloids scar will result in cosmetic disfigurement, functional impairment, and affect the quality of life. Triamcinolone acetonide (TAC) is traditionally employed in treating keloid scars. In this study, we aim to evaluate the effectiveness of TAC and compare it with other common therapy employed in keloid treatment. Only randomized controlled trial (RCT) and controlled trial were included. Inverse variance risk ratio, weighted mean difference, and corresponding 95% confidence intervals were calculated to evaluate the effect of intervention. Meta-analysis indicated that TAC treatment significantly reduced the size of keloid compared to untreated control. Reduction in size was statistically different in favor of TAC compared to silicone gel sheet. Significant difference in favor of TAC was observed compared with verapamil in term of vascularity and scar pliability. TAC treatment was more effective in reducing scar thickness in comparison with cryotherapy. However, the current meta-analysis has several limitations. Only a limited number of trials with the same comparison are available. Most trials recruited a small number of patients and used inconsistent outcome assessment. Most trials did not provide detail information on allocation concealment and blinding. Therefore, further evaluation in multi-center RCTs with consistent comparisons and outcome measurements are warrant to reach a consensus on the selection between TAC and different treatment modalities.

## Introduction

Keloid is a cutaneous dermal lesion resulting from uncontrolled deposition of collagen and glycosaminoglycan around the wound. Elevated levels of growth factor and cytokines contribute to keloid formation (1–3). Transforming growth factor beta (TGF-β) family is associated with enhanced collagen synthesis in keloid fibroblasts. TGF-β1 treatment stimulates the production of collagen in keloid fibroblasts but not in normal skin fibroblasts (1). Observation that anti-TGF-β1 antibody suppresses collagen synthesis of keloid fibroblasts further confirms the role of TGF-β1 (1). TGF-β2 treatment enhances collagen production of xenograft derived from human keloid specimens in athymic rats, indicating a causative role of TGF-β2 in keloid formation (2). In contrast, TGF-β2 antibody inhibits collagen generation in xenograft model, suggesting that it could act as a potential antiscarring agent (2). Interleukin (IL)-13 induces a more rapid increase in collagen generation in keloid fibroblasts compared to normal fibroblasts (3).

Keloid could grow spontaneously or grow following dermal trauma with poor prognosis. The uncontrolled growth of keloid will continue to grow without regression, and the patients will experience itch, pruritis, and pain. Common occurring sites include chest, shoulder, earlobes, and upper back (4). When the fibrous keloid become big, it will lead to cosmetic disfigurement, functional impairment, and affect the quality of life (4). Since keloid has notoriously high recurrence rate after surgical excision, non-surgical means are recommended for the primary keloid treatment (5). Non-surgical means include corticosteroids injection, 5-fluorouracil (5-FU), verapamil, silicon gel sheets, cryotherapy, pulsed dye laser (PDL), and radiation. Intralesional injection of corticosteroid triamcinolone acetonide (TAC) is one of the first-line treatment modalities for keloid treatment (5). Corticosteroid is highly tolerated by the patients with a keloid. Corticosteroid could diminish the exuberant collagen synthesis and inhibit the rapid growth of keloid fibroblasts (6). In addition, corticosteroid could promote vasoconstriction in the keloid scar and control local inflammation (7). However, it is also noticed that the response rate of TAC treatment is highly varying with high recurrence rate (4, 6). TAC monotherapy may induce hypopigmentation, mixed pigmentation, fat atrophy, telangiectasias, necrosis, ulcerations, and cushingoid habitus (8). In addition, there are concern on the repeated use of corticosteroid at high-dose in patients with large and multiple keloids (4). 5-FU functions by inhibiting the synthesis of pyrimidine thymidine and interferes the DNA replication process in the rapidly dividing cells by competing with uracil (9). Verapamil functions by regulating the balance between fibroblasts and extracellular matrix remodeling (10). Silicon gel sheets could act as an occlusive layer which suppresses IL-1 and IL-6 production, thus inhibiting fibroblast synthesis (11). Cryotherapy could destruct the keloid by the formation of sharp ice crystals and the induction of ischemic necrosis (12). PDL promotes keloid regression by photothermolysis (13). The light energy emitted by PDL causes coagulation necrosis of fibroblasts. PDL also suppresses proliferation and triggers apoptosis of fibroblasts. Radiation suppresses proliferation of fibroblasts, leading to a reduction in collagen generation (14).

At present, there is still no consensus of the effectiveness between different treatment modalities and their effectiveness remains controversial. The aim of the current study is to evaluate the efficacy of TAC-based therapy for keloid treatment. In addition, we asked whether combined treatment with TAC and other treatment modalities is superior to TAC alone. Finally, the effectiveness of TAC-based treatment versus treatment regimes with the use of 5-FU, verapamil, silicon gel sheeting, or cryotherapy will also be performed.

## Materials and Methods

### Search Strategy

A systematic literature retrieval was performed in different databases including PubMed, EMBASE, and MEDLINE using the search terms (((((triamcinolone) OR corticosteroids) OR steroids)) AND ((((((randomised controlled trials) OR controlled clinical trials) OR controlled clinical trial) OR randomised controlled trial) OR clinical trial) OR clinical trials)) AND ((((hypertrophic) AND ((((scar) OR scars) OR scarred) OR scarring))) OR keloid). Full articles and abstract are all included. The search was performed in April 2016.

### Study Selection

Two reviewers assessed the eligible trials independently. Only randomized controlled trial (RCT) and controlled trial (CT) on patients with pathological confirmed keloid were included. Studies that compared the regime of TAC with a non-steroid-based treatment modality were included.

### Primary and Secondary Outcomes

The primary outcomes included (1) reduction in scar height, thickness, size, vascularity, pliability, and pigmentation and (2) overall scar improvement obtained from patient self-assessment and observer assessment. Patient self-assessment was graded by patients with a 5-point scale: no improvement; poor, up to 25% improvement; fair, 26–50%; good, 51–75% improvement; and excellent, 76–100% improvement. Observer assessment was graded by the observer using a scale that was the same as patient self-assessment. Over 50% improvement was regarded as effective. The secondary outcome was improvement in erythema, pain, and itch. Adverse events included hypopigmentation, telangiectasia, and skin atrophy.

### Data Extraction

Two reviewers extracted data independently. Study characteristics (author, year of publication, country, number of patients, intervention and control, follow-up period, primary and secondary outcomes) and participant characteristics (age and sex) were summarized. The numbers of patients with >50% improvement, >50% reduction in size, or any specific adverse event were extracted from both arms of intervention. The values of Vancouver Scar Scale including scar pigmentation, vascularity, pliability, and height were extracted from both groups. The values of scar thickness before and after intervention were also extracted.

### Evaluation of Risk of Bias

The risk of bias was evaluated according to Cochrane handbook and analyzed by Review Manager 5.3. The parameters included random sequence generation, allocation concealment, blinding, incomplete outcome data, and selective reporting.

### Statistical Analysis

For dichotomous variables, inverse variance risk ratio (RR) and corresponding 95% confidence intervals (CI) were calculated to evaluate the effect of intervention. For continuous data comparisons, weighted mean difference (WMD) and 95% CI were calculated. Heterogeneity between studies was assessed using *p* value of *I*^2^ statistics. *p* value of *I*^2^ statistics smaller than 0.1 was classified as indication of substantial heterogeneity. Where heterogeneity was observed, random effects model was used. In contrast, fixed effect model was adopted. For TAC treatment, different dosages of TAC were used. A subgroup analysis was performed according to the dosage of TAC. All the analysis was carried out using STATA version 12.0 (StataCorp). Unless otherwise specified, *p* value below 0.05 was denoted as statistical significance.

## Results

### Paper Selection and Study Characteristics

**Figure 1:**
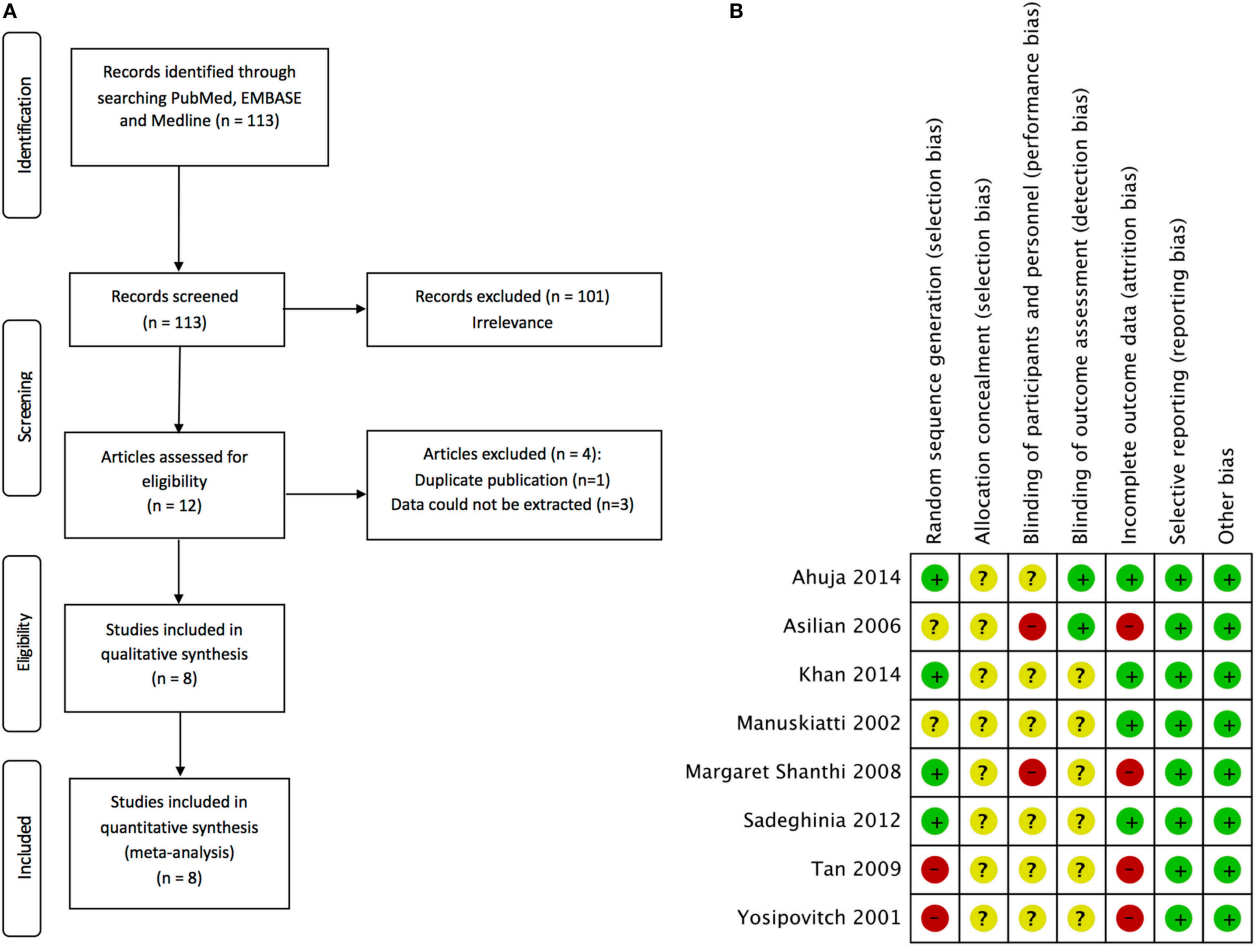
**Schematic representation of the search strategy and risk of bias summary of selected studies**. **(A)** Flow chart showing the selection process of the studies in the meta-analysis. **(B)** review authors’ judgments about each risk of bias item for each included study. +, low risk; −, high risk; ?, unclear risk.

A total of 113 studies were identified by searching Pubmed, EMBASE, and Medline. After excluding 101 irrelevant studies, 12 studies were selected for further evaluation and all the studies were available in full paper (Figure 1A). Four studies were further excluded due to duplicate publication or no data on defined primary and secondary outcomes available. The study of Darougheh et al. (15) has duplicate data with Asilian et al. (16); therefore, it was excluded for meta-analysis. No data on reduction in scar size or overall scar improvement could be extracted from the studies of Layton et al. (17), Sproat et al. (18), and Kelemen et al. (19). Finally, eight studies were included in this meta-analysis (11, 16, 20–25). The characteristics of these eight studies were shown in Table 1.

**Table 1:** **Characteristics of the studies selected in this meta-analysis**.

Author	Year	Country	Type of assessment	Age (years)	Gender (male/female)	No. of patients (arm 1)	No. of patients (arm 2)	Follow-up	Treatment in arm 1	Treatment in arm 2
**Triamcinolone acetonide (TAC) versus control**										
Tan et al. (11)	2009	Singapore	Reduction in size	19–40	18/2	20 (TAC)	20 (Control)	12 weeks	TAC 40 mg/ml	No treatment

**TAC versus silicone gel sheet**										
Tan et al (11)	2009	Singapore	Reduction in size	19–40	18/2	20 (TAC)	20 (Silicone gel sheet)	12 weeks	TAC 40 mg/ml	Silicone gel sheet

**TAC versus 5-fluorouracil (5-FU)**										
Sadeghinia and Sadeghinia (22)	2012	Iran	Patient self-assessment	NA	NA	20 (TAC)	20 (5-FU)	44 weeks	TAC 40 mg/ml	5-FU 50 mg/ml
Manuskiatti and Fitzpatrick (21)	2002	Thailand	Patient self-assessment	25–74	4/6	10 (TAC)	10 (5-FU)	32 weeks	TAC 20 mg/ml	5-FU 50 mg/ml

**TAC versus TAC + 5-FU**										
Asilian et al. (16)	2006	Iran	Patient self-assessment	5–70	15/25	20 (TAC)	20 (TAC + 5-FU)	12 weeks	TAC 10 mg/ml	TAC 4 mg/ml + 5-FU 45 mg/ml
Khan et al. (23)	2014	Pakistan	Observer assessment	NA	65/85	75 (TAC)	75 (TAC + 5-FU)	12 weeks	TAC 10 mg/ml	TAC 4 mg/ml + 5-FU 45 mg/ml
Manuskiatti and Fitzpatrick (21)	2002	Thailand	Patient self-assessment	25–74	4/6	10 (TAC)	10 (TAC + 5-FU)	32 weeks	TAC 20 mg/ml	TAC 1 mg/ml + 5-FU 45 mg/ml

**TAC versus verapamil**										
Margaret Shanthi et al. (24)	2008	India	Vancouver scar scale	NA	NA	27 (TAC)	27 (Verapamil)	24 weeks	TAC 40 mg/ml	Verapamil 2.5 mg/ml
Ahuja and Chatterjee (25)	2014	India	Vancouver scar scale	15–60	NA	22 (TAC)	26 (Verapamil)	24 weeks	TAC 40 mg/ml	Verapamil 2.5 mg/ml

**TAC versus cryotherapy**										
Yosipovitch et al. (20)	2001	Singapore	Scar thickness	17–50	NA	10 (TAC)	8 (Cryotherapy)	4 weeks	TAC 40 mg/ml	Cryotherapy

**TAC versus TAC + cryotherapy**										
Yosipovitch et al. (20)	2001	Singapore	Scar thickness	17–50	NA	10 (TAC)	10 (TAC + cryotherapy)	4 weeks	TAC 40 mg/ml	TAC 40 mg/ml + cryotherapy

*NA, not available*.

### Risk of Bias in Included Studies

The risk of bias of eight studies included in this meta-analysis was summarized in Figure 1B. Since the studies of Yosipovitch et al. (20) and Tan et al. (11) were not RCT, they were judged as high risk for random sequence generation. Four studies (22–25) provided the method used to generate random sequence and were judged to be at low risk of random sequence generation. Two studies (16, 21) did not report the method of generating random sequence and we judged them at unclear risk. Allocation concealment was not reported in all of the eight studies (11, 16, 20–25), and we judged all trials as unclear for allocation concealment. Two single-blinded trials (16, 24) were judged as high risk for blinding of participants and personnel. Complete information on the blinding processes was not shown by the left six studies (11, 20–23, 25) and we judged them at unclear risk for performance bias. Two studies (16, 25) employed blinded observer to assess outcomes and were judged at low risk of detection bias. The left six trials (11, 20–24) were judged as unclear risk due to no report on blinding of outcome assessment. Four trials (11, 16, 20, 24) were judged at high risk for incomplete outcome data because the numbers lost to follow up were high and no reasons were given for the losses. No reporting bias or other bias was observed in all eight studies (11, 16, 20–25). In summary, most studies have no detail information on allocation concealment, blinding of participants and personnel, and blinding of outcome assessment.

### TAC versus Control

Tan et al. (11) evaluated the efficacy of TAC in keloid treatment. A total of 20 patients with multiple keloids were recruited and treated by TAC for 12 weeks. Seventeen patients in both TAC group and control group completed the 12-week treatment. A significant difference was observed in >50% reduction in size (RR: 33.00, 95% CI: 2.14–509.33) and improvement in erythema (RR: 21.00, 95% CI: 1.33–332.06), favoring TAC in comparison with control. In terms of improvement in pain and itch, there was no statistically significant difference. These results indicated that TAC treatment significantly reduced the size and improved appearance of keloid.

### TAC versus Silicone Gel Sheet

Tan et al. (11) compared the effectiveness of TAC with silicone gel sheet in keloid treatment. Seventeen patients in both TAC group and silicone gel sheet group completed the 12-week treatment. The >50% reduction in size (RR: 8.00, 95% CI: 2.16–29.57) and improvement in erythema (RR: 10.00, 95% CI: 1.43–69.77) were statistically different in favor of TAC compared to silicone gel sheet. For improvement in pain and itch, no apparent difference was observed. TAC treatment not only resulted in a decrease in size but also normalized symptoms of keloid in comparison with silicone gel sheet.

### TAC versus 5-FU

The efficiency of TAC was compared with 5-FU in two trials (21, 22). In the study of Manuskiatti and Fitzpatrick (21), 10 patients including 6 women and 4 men with age ranging from 25 to 74 were treated by TAC or 5-FU. Over 50% improvement was observed in 10 out of 10 patients in both TAC arm and 5-FU arm. The RR was 1 with the standard error of the log relative risk to be 0 and 95% CI was 1 to 1. In data synthesis, this study was excluded by STATA. Sadeghinia and Sadeghinia (22) compared the effects of TAC and 5-FU using patient self-assessment. Forty patients were randomized into TAC group (20 patients) or 5-FU group (20 patients). The ratio of patients with >50% overall improvement was compared. A significant improvement in favor of 5-fluorouracil compared to those treated with TAC (RR: 2.12, 95% CI: 1.20–3.75). The study of Sadeghinia and Sadeghinia (22) showed that no side effect was detected in both TAC and 5-FU group. In contrast, Manuskiatti and Fitzpatrick (21) reported that skin atrophy, telangiectasia, and hypopigmentation were noted in 50% segments of TAC group but not in 5-FU group. However, the difference in complications between TAC and 5-FU groups was not statistically significant (RR: 11.0, 95% CI: 0.69–175.86).

### TAC versus TAC with 5-FU

**Figure 2:**
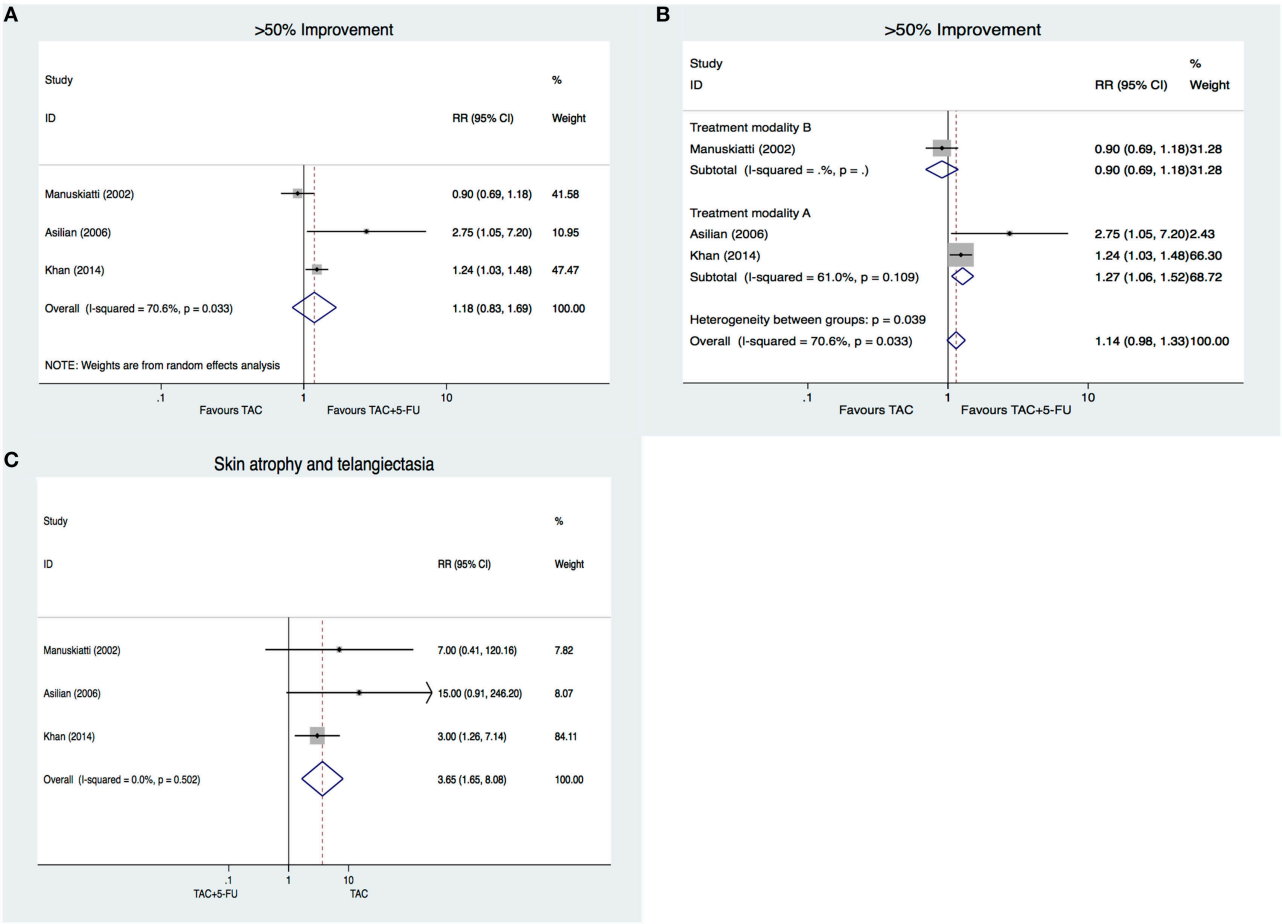
**Triamcinolone acetonide (TAC) versus TAC with 5-FU in keloid treatment**. **(A)** Forest plot representing difference in over 50% improvement between TAC and TAC with 5-FU. **(B)** Effects of treatment regime on improvement. **(C)** Complications of TAC and TAC with 5-FU.

Three trials compared the effects of TAC alone with the combination of TAC and 5-FU (16, 21, 23). There are two outcomes including patient self-assessment and observer assessment. Over 50% improvement was regarded as effective. There was no significant difference between the two groups (RR: 1.18, 95% CI: 0.83–1.69; Figure 2A). For TAC treatment, there are two different regimes: (A) TAC 10 mg/ml, once weekly, eight sessions; and (B) TAC 20 mg/ml, once every 4 weeks, six sessions. For the combination treatment using TAC and 5-FU, TAC dosage reduced to 1–4 mg/ml together with 45 mg/ml 5-FU. Except Manuskiatti and Fitzpatrick (21) (regime B, 20 mg/ml), all other studies used the same treatment regime (regime A, 10 mg/ml). When we performed subgroup analysis based on the treatment regime, a statistically significant difference in favor of TAC with 5-FU was only observed in the studies using 10 mg/ml TAC (RR: 1.27, 95% CI: 1.06–1.52; Figure 2B) but not in the study using 20 mg/ml TAC (RR: 0.90, 95% CI: 0.69–1.18; Figure 2B). In terms of complications, patients in TAC group have a higher risk to experience skin atrophy and telangiectasia compared with combination treatment using TAC and 5-FU (RR: 3.65, 95% CI: 1.65–8.08; Figure 2C). TAC plus 5-FU showed advantages over TAC alone only when a low dose of TAC was used in TAC monotherapy.

### TAC versus Verapamil

**Figure 3:**
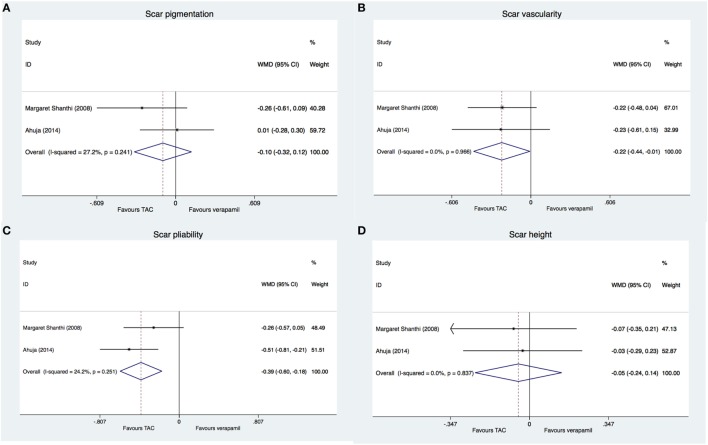
**Comparison of triamcinolone acetonide (TAC) with verapamil in the treatment of keloid**. Forest plot showing differences in pooled scar pigmentation **(A)**, vascularity **(B)**, pliability **(C)**, and height **(D)** between TAC and verapamil treatment.

Two trials (24, 25) compared the use of TAC with verapamil. Forty patients (48 scars) were divided into TAC group (22 scars) and verapamil group (26 scars) in the study of Ahuja and Chatterjee (25). In the trial of Margaret Shanthi et al. (24), 54 patients were allocated to TAC arm (27 patients) and verapamil arm (27 patients). In both trials, Vancouver Scar Scale including scar pigmentation, vascularity, pliability, and height was used to measure the treatment effects. For scar pigmentation, no statistical difference between TAC and verapamil was observed at 3 weeks (WMD: −0.10, 95% CI: −0.32–0.12; Figure 3A); when vascularity was used as outcome, there was a statistically significant difference in favor of TAC compared to verapamil at 3 weeks (WMD: −0.22, 95% CI: −0.44 to −0.01; Figure 3B); in terms of scar pliability, significant improvement in favor of TAC was observed after 3 weeks (WMD: −0.39, 95% CI: −0.60 to −0.18; Figure 3C); for scar height, no statistical difference between TAC and verapamil was noticed (WMD: −0.05, 95% CI: −0.24–0.14; Figure 3D). Although Margaret Shanthi et al. (24) reported that complications were seen in both groups, no data were presented. In the trial of Ahuja and Chatterjee (25), the difference in complications including skin atrophy and telangiectasia was not statistically significant between TAC and verapamil groups (RR: 15.26, 95% CI: 0.91–256.58). TAC treatment improved the symptoms of keloid but not reduced the height of keloid compared to verapamil.

### TAC versus Cryotherapy

The effectiveness of TAC was compared with cryotherapy by Yosipovitch et al. (20). Fourteen patients with at least two keloids aged between 17 and 50 years were enrolled in this study. The WMD in scar thickness was statistically different in favor to TAC (WMD: −3.35, 95% CI: −4.95 to −1.75).

### TAC versus TAC Plus Cryotherapy

Yosipovitch et al. (20) compared the efficacy between TAC and TAC plus Cryotherapy. The WMD in scar thickness was statistically different in favor to TAC plus cryotherapy (WMD: −4.60, 95% CI: −7.54 to −1.66).

## Discussion

Keloid is an excessive scar with high tendency to extend beyond the initial wound margin and persistent long without spontaneous regression (6). Surgical manipulation is not the best option for keloid management as the recurrence rate is high (5). Alternative therapies using corticosteroids injection, chemotherapeutic agents, verapamil, silicon gel sheets, and cryotherapy become important especially in case where patients presented with high recurrence rate after surgery (4). Selection between different treatment modalities is usually experience based and the varying success rates were obtained. Khansa et al. performed a literature review to provide evidence-based evaluation on the effectiveness of different treatment methods for keloid (26). Treatment modalities including silicone gel, PDL, TAC, and 5-FU showed high efficacy in improving keloid. In contrast, onion extract and fat grafting exhibited low efficiency (26). In this study, we further compared the effectiveness of TAC with different treatment regimes.

Although intralesional injection of TAC is one of the first-line treatments for keloid, prospective CTs comparing its efficacy with different treatment modalities remained few and presented with different outcomes (27). Results from current meta-analysis showed that TAC treatment resulted in marked reduction in the size of keloid in comparison with untreated control. TAC was more effective in improving scar than silicone gel sheet, verapamil and cryotherapy. In addition, 5-FU showed a significant improvement of keloid in comparison with TAC. Although TAC treatment could lead to complications including skin atrophy and telangiectasia, the difference was not statistically significant compared to 5-FU or verapamil.

Bijlard et al. (28) performed an up-to-date review on the RCTs, prospective clinical trials, and case series involving keloid treatment using intralesional 5-FU alone or in combination. In general, keloid treated with 5-FU showed good outcome with reduced pain and pruritis. However, patients may experience adverse event accompanied with the used of chemotherapeutic drug such as pain at injection site, ulceration, and burning sensation (28). It should also be noted that the use of 5-FU in keloid treatment has multiple limitations. Although the use of 5-FU at high-dose without the presentation of hematological complication is reported, the use should be cautious in cases of pregnancy, lactation, intercurrent infection, and bone marrow depression (29).

The use of TAC together with the chemotherapeutic drug 5-FU is increasing. In the study of Shah et al., 5-FU in conjunction with TAC displayed the highest efficiency in improving the symptoms of scars and decreasing recurrence (30). Our subgroup analysis revealed that TAC in combination with 5-FU was more effective than TAC alone only when TAC was used at a low concentration (10 mg/ml). The combination treatment was not superior to TAC alone when 20 mg/ml TAC was employed. In order to achieve keloid resolution, TAC at high concentration (40 mg/ml) is recommended for TAC monotherapy (31). Thus, we could not conclude that TAC plus 5-FU offers a better treatment outcome in comparison with TAC. In addition, the effects of TAC in the combination treatment is difficult to assess as all authors reduced the TAC concentration (from 10–20 mg/ml to 1–4 mg/ml) when used in combination with 5-FU. Usually, the use of TAC in the combination treatment is not for therapeutic intent. It is used for the potential inflammation events accompanied with the 5-FU treatment (29). However, TAC plus 5-FU exhibited lower complications compared with TAC alone.

Verapamil is a calcium antagonist which could induce procollagenase production leading to the reduction in collagen production in scar fibroblasts (7). The calcium channel blocker could induce a phenotypic change in fibroblast from bipolar shape to spheroidal shape by depolymerizating the actin filament (32). Verapamil triggers extracellular matrix remodeling by preventing tritiated proline incorporation (33). The use of verapamil in patients with burn scars is considered to be safe and cost-effective. Lawrence (34) was the first group who employed the use of verapamil in earlobe keloid treatment. They showed that 52% keloid patients were cured when intralesional verapamil (2.5 mg/ml) was administered at 7–14 day after keloid removal. Verapamil was administered at 1-month interval where feasible. In comparison with TAC, there was a significant improvement in favor of TAC if we used scar vascularity (at 3 weeks) and scar pliability (at 3 weeks) as outcomes. In contrast, when scar pigmentation and height were used, no statistical significant difference was observed. These results indicated that TAC reduced the scar vascularity and pliability faster than verapamil.

TAC is widely used as first-line therapy in treating keloid by practitioners. However, its functional mechanism is less clear in comparison with other treatment modalities examined in the current study. Given that TAC treatment will lead to substantial side effects, selection between TAC and other treatment options should be evidence based and the discernible benefits shall be clarified. Observation that TAC treatment is more effective in improving keloid in comparison with silicone gel sheet, verapamil, and cryotherapy promotes us to recommend TAC for keloid treatment. In light of that TAC in combination with 5-FU has reduced complications in comparison with TAC monotherapy, TAC in conjunction with 5-FU should be recommended for keloid treatment.

There are several limitations in our systematic reviews. We found that there are no sufficient research trials comparing TAC and other treatment options. The trials performed using inconsistent outcome assessment with varying follow-up period. No data on recurrence are available in most trials. Given that keloid is a disease with high recurrence rate, comparison between TAC and other treatment modalities in preventing keloid recurrence shall be performed. For the dosage of TAC injection, most study used TAC at 40 mg/ml. However, there are not much data on the injection volume and keloid size. In addition, most trials only recruit a small number of patients with keloid at different anatomical locations. For the dermatological examination, Vancouver Scar Scale (evaluation of vascularity, pigmentation, pliability, and height), observer assessment, and self-assessment were used at different follow-up duration with no information on the blinded assessment. Further evaluation in multi-center RCTs with consistent objective, repeatable outcome, and recurrence measurement are warrant to reach a consensus on the selection between TAC and different treatment modalities.

## Author Contributions

WG and T-SW conceived the study. T-SW, JL, and SC reviewed and extracted data from the literature. JL, SC, JC, and WG performed the meta-analysis and interpretation of the data. T-SW, JC, and WG drafted and revised the manuscript. All authors red and approved the final manuscript.

## Conflict of Interest Statement

The authors declare that the research was conducted in the absence of any commercial or financial relationships that could be construed as a potential conflict of interest.
